# Wisdom of the Experts Versus Opinions of the Crowd in Hospital Quality Ratings: Analysis of Hospital Compare Star Ratings and Google Star Ratings

**DOI:** 10.2196/34030

**Published:** 2022-07-26

**Authors:** Hari Ramasubramanian, Satish Joshi, Ranjani Krishnan

**Affiliations:** 1 Accounting Department Frankfurt School of Finance and Management Frankfurt am Main Germany; 2 College of Agriculture & Natural Resources Department of Agricultural, Food, and Resource Economics Michigan State University East Lansing, MI United States; 3 Accounting and Information Systems Broad College of Business Michigan State University East Lansing, MI United States

**Keywords:** hospital quality, web-based rating, online ratings, Hospital Compare, star ratings

## Abstract

**Background:**

Popular web-based portals provide free and convenient access to user-generated hospital quality reviews. The Centers for Medicare & Medicaid Services (CMS) also publishes Hospital Compare Star Ratings (HCSR), a comprehensive expert rating of US hospital quality that aggregates multiple measures of quality. CMS revised the HCSR methods in 2021. It is important to analyze the degree to which web-based ratings reflect expert measures of hospital quality because easily accessible, crowdsourced hospital ratings influence consumers’ hospital choices.

**Objective:**

This study aims to assess the association between web-based, Google hospital quality ratings that reflect the opinions of the crowd and HCSR representing the wisdom of the experts, as well as the changes in these associations following the 2021 revision of the CMS rating system.

**Methods:**

We extracted Google star ratings using the Application Programming Interface in June 2020. The HCSR data of April 2020 (before the revision of HCSR methodology) and April 2021 (after the revision of HCSR methodology) were obtained from the CMS Hospital Compare website. We also extracted scores for the individual components of hospital quality for each of the hospitals in our sample using the code provided by Hospital Compare. Fractional response models were used to estimate the association between Google star ratings and HCSR as well as individual components of quality (n=2619).

**Results:**

The Google star ratings are statistically associated with HCSR (*P*<.001) after controlling for hospital-level effects; however, they are not associated with clinical components of HCSR that require medical expertise for evaluation such as safety of care (*P*=.30) or readmission (*P*=.52). The revised CMS rating system ameliorates previous partial inconsistencies in the association between Google star ratings and quality component scores of HCSR.

**Conclusions:**

Crowdsourced Google star hospital ratings are informative regarding expert CMS overall hospital quality ratings and individual quality components that are easier for patients to evaluate. Improvements in hospital quality metrics that require expertise to assess, such as safety of care and readmission, may not lead to improved Google star ratings. Hospitals can benefit from using crowdsourced ratings as timely and easily available indicators of their quality performance while recognizing their limitations and biases.

## Introduction

In recent years, crowdsourced, web-based information aggregated by social media platforms, service providers, and government agencies has become a popular source for consumers, organizations, and governments. If used effectively, crowdsourcing can gather timely feedback, improve efficiency and supervision, reduce response times for critical actions, and increase customer engagement and satisfaction [1]. Although crowdsourced ratings are widely used, including in health care settings, concerns arise about crowdsourced assessments due to the following reasons: (1) nonrepresentative nature of the sampling process, (2) inadequacies in expertise required to assess the quality of the goods or services provided, (3) the limited nature of experiences, and (4) the potential for manipulation [1-4]. Especially for complex services such as medical treatment, consumers may not have the expertise to evaluate the quality of care even after the completion of the consumption cycle, and crowdsourced ratings may have little or no correspondence with true quality, due to cognitive limitations and expertise gap [5].

In this analysis, we explored the association between a popular crowdsourced, web-based hospital quality rating (Google star) and expert rating of hospital quality (HCSR) published by the Centers for Medicare & Medicaid Services (CMS) [6]. Understanding the association between crowdsourced opinions and expert hospital quality ratings is important because web-based ratings appear to have substantial influence on patients’ health care choices [7]. For example, 65% of surveyed patients in 2020 used web-based reviews to evaluate doctors, and Google was the most visited review site [8,9]. In comparison, only 22% of the surveyed patients were even aware of CMS’s expert ratings [10].

While the Hospital Compare website provides information on more than 100 quality measures for more than 4000 US hospitals, HCSR aggregates multiple quality measures into a single overall star rating to increase user convenience. CMS revised the HCSR methodology in 2021. Previously, 57 measures of hospital quality were assigned to the 7 clusters of hospital quality, namely, “patient experience,” “mortality,” “readmission,” “safety of care,” “efficient use of medical imaging,” “timeliness of care,” and “effectiveness of care,” and a hospital-specific score was derived for each of these groups using a latent variable statistical model. Hospital summary score was computed as a weighted sum of its group scores, and hospitals were assigned to the star categories based on a clustering algorithm [11-13]. The revised HCSR method reduced the number of quality measures to 48 and quality clusters to 5 by combining the separate measures for “timeliness of care”, "effectiveness of care", and “efficient use of medical imaging.” The latent variable model was replaced by a simple average of individual measures making the scores transparent, understandable, and predictable [12].

We empirically analyzed the associations between Google star and HCSR before and after the revisions. We also analyzed the associations with HCSR quality component scores. The analyses reveal that Google star ratings are statistically associated with HCSR (*P*<.001) after controlling for hospital-level effects but are not associated with clinical components of HCSR that require medical expertise for evaluation, such as safety of care (*P*=.30) or readmission (*P*=.52), after the 2021 HCSR revision.

While a number of prior studies have analyzed the relationship between crowdsourced hospital ratings (eg, Yelp and Facebook) and expert ratings [14-17], our study contributes to the literature by examining the association between the more popular Google star ratings and expert HCSR ratings before and after the recent revision of HCSR methodology. Unlike the previous research, which has analyzed associations with some selected clinical quality indicators [18,19], we analyzed the associations with all the individual HCSR quality cluster scores. Additionally, we analyzed these associations by employing a more refined statistical technique of fractional response modeling that accounts for boundedness and the nonlinear nature of the star ratings while controlling for other covariates [20].

## Methods

### Data Source

We used the National Bureau of Economic Research’s Healthcare Cost Report Information System to generate a data set of 4615 US hospitals and their characteristics such as the size (number of beds), type in terms of location (rural or urban), and status, that is, for-profit or nonprofit and teaching or nonteaching. We collected HCSR data from the CMS Hospital Compare website in April 2020 before the revision of HCSR, and again in April 2021 after the revision. We also extracted scores for the individual components of hospital quality for each of the hospitals in our sample using the code provided by CMS. In June 2020, we extracted Google star ratings for all available US hospitals using the Application Programming Interface, which is a standard interface for collecting data from web-based portals. Google Places Application Programming Interface provides Google’s Star ratings, which are the cumulative average of consumer ratings of each hospital, ranging between 1 and 5. To assure quality, Google removes all anonymous reviews, requires a valid associated email address, and does not allow more than one review per business from a particular email [21]. Because the 2020 HCSR draw on hospital quality data covering the period 2015-2018, and the 2021 HCSR use data for the period 2016-2019, we used the Google star ratings extracted in June 2020, which are cumulative ratings and useful for analyzing associations both before and after the HCSR revision. This facilitated the consistent analysis of these associations, especially in view of the potential impact of the COVID-19 pandemic on subsequent Google star hospital ratings. A total of 2963 US hospitals had received HCSR and all the component scores in both 2020 and 2021 reporting cycles, and the 46 (1.6%) hospitals that did not have Google star ratings were excluded from analyses. To ensure that Google star ratings were representative, and following a similar approach used in previous studies [14], we excluded 298 (10.1%) hospitals, which had less than 10 individual Google ratings. This resulted in the final sample of 2619 US hospitals with an average of 179 individual Google ratings per hospital. However, including hospitals with less than 10 individual Google ratings in our estimations resulted in similar conclusions.

### Research Design

We theorize that changes in true hospital quality influence both HCSR expert ratings and crowdsourced, web-based Google star ratings, and as a result, HCSR and Google star ratings will be correlated. However, hospital-level characteristics such as size and type are also likely to affect consumer perceptions of hospital quality and therefore web-based ratings. Unlike HCSR that factors in hospital-level drivers of quality or risk, Google star ratings are not adjusted for hospital characteristics. Therefore, it is important to control for these while assessing associations between Google star and HCSR. Further, improvements in health quality component scores improve hospital quality and, by design, their aggregate HCSR; we thus hypothesize that crowdsourced Google star ratings will also be associated with HCSR quality component scores. While it would be ideal to explore the association of HCSR component scores with similar quality component scores in Google star ratings (which are not available) or some proxies for quality clusters developed from detailed textual analyses of accompanying comments, we posit that associations between aggregate Google star ratings and HCSR component scores will be informative approximations.

We estimated the following models to examine the association between Google hospital ratings, HCSR, and individual HCSR component scores, after controlling for hospital size and hospital type effects on web-based ratings.



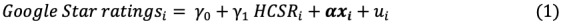





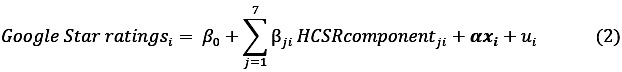



Where 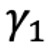 is the association between Google star ratings and HCSR, 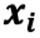 is a vector of control variables for hospital *i*, which includes size (logarithm of the number of beds), and hospital type (for-profit, not-for-profit, or government hospital; teaching or nonteaching hospital) and their interaction terms. *HCSRcomponent_j_* is the HCSR component score for each cluster of hospital quality. Our dependent variable, Google star ratings, is a continuous variable bounded between 1 and 5, and linear estimation methods can produce predicted values outside these bounds. Therefore, we scaled the Google star ratings to be bounded between 0 and 1 and employ a fractional response model (FRM) to estimate these equations [20]. The FRM is an extension of the generalized linear model that accounts for the boundedness of the dependent variable from both above and below, predicts response values within the interval limits of the dependent variable, and captures the nonlinearity of the data, thereby yielding a better fit compared to linear estimation models. Furthermore, the FRM does not require special data transformations at the corners; it also permits a robust, consistent, and relatively efficient estimation of the conditional expectation of the dependent variable given the predictors [20].

## Results

**Figure 1:**
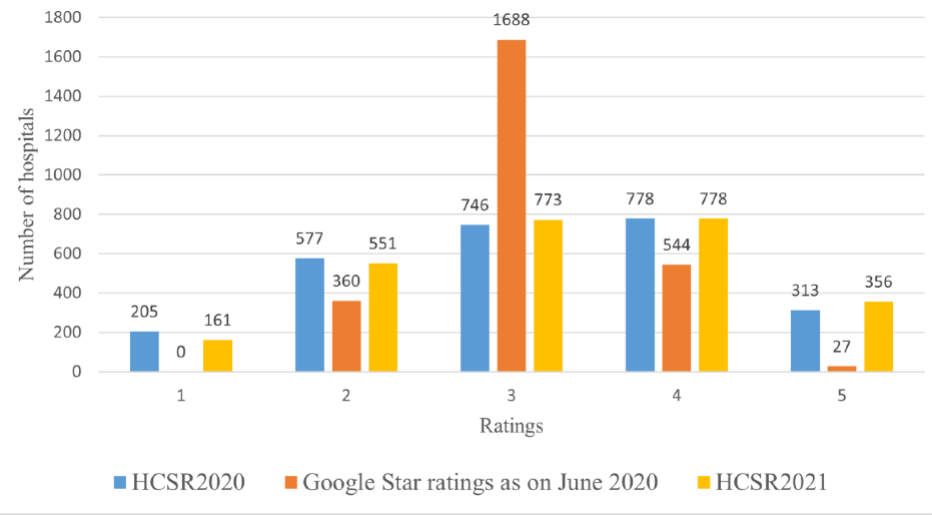
Graph showing HCSR2020, HCSR2021, and Google Star ratings for the sample of 2619 US hospitals. HCSR: Hospital Compare Star Ratings.

Figure 1 shows the distributions of “HCSR2020,” “HCSR2021” (the Hospital Compare Star ratings provided by CMS in April 2020 and April 2021, respectively) and “Google star” ratings. The means of HCSR2020, HCSR2021, and Google star ratings are similar with values of 3.159, 3.236, and 3.040, respectively. However, the distributions of HCSR are relatively wider when compared to Google star ratings, as evidenced by standard deviations of 1.133, 1.114, and 0.557, respectively. In Figure 1, Google star ratings have been rounded up or down to the nearest integer in the bar graph. Accordingly, HCSR2020, HCSR2021, and Google star ratings in the graph can take integer values from 1 and 5.

Simple correlation analyses show statistically significant (*P*<.001) Pearson correlation coefficients of 0.234 between Google star ratings and HCSR2020, and 0.226 between Google star and HCSR2021 ratings. Spearman correlation coefficients are 0.242 between Google star and HCSR2020, and 0.224 between Google star and HCSR2021, and both are significant (*P*<.001).

Since the above results do not control for variations in hospital characteristics, we estimate the parameters of Equation (1) employing FRM regression techniques with scaled Google star ratings as a dependent variable; we also estimate HCSR as well as hospital size and type as explanatory variables. Table 1 reports the estimation results for the relationship between Google star ratings and Hospital Compare Star Ratings (HCSR). The dependent variable is Google star ratings scaled by 5 to be between 0 and 1. The results are estimated using fractional response probit regression with standard errors robust to distributional misspecification. The coefficients on HCSR2020 and HCSR2021 are 0.076 and 0.070 and statistically significant (*P*<.001). The statistically significant coefficient estimates on HCSR confirm that Google star ratings have informative value even after controlling for hospital factors. Similarly, significant coefficients on hospital size and type variables (eg, for-profit, teaching, rural, and their interactions) support our hypothesis that hospital characteristics influence consumer perceptions of hospital quality.

Table 2, column 1 shows FRM estimates of the association between HCSR2020 and its individual components, while column 2 shows FRM estimation results (Equation 2) with Google star ratings as the dependent variable and HCSR2020 component scores of hospital quality as explanatory variables, with controls for hospital size and type. Columns 3 and 4 show similar estimation results using the component scores for HCSR2021. In all the columns, dependent variable is scaled by dividing by 5 to be between 0 and 1. The results are estimated using fractional response probit regression with standard errors robust to distributional misspecification.

As can be seen from columns 1 and 3, both HCSR2020 and HCSR2021 are positively associated with all the component scores (*P*<.001), which is unsurprising since overall HCSR is assigned based on the weighted sum of individual scores. The results in column 2 show that Google star ratings had statistically significant positive association with patient experience (*P*<.001), mortality (*P*<.001), and effectiveness of care (*P*<.001) components of HCSR2020. Google star ratings were negatively associated with the 2020 quality scores for readmission (*P*=.01) and efficient use of medical imaging (*P*=.06), indicating that hospitals considered by experts as high performing on these 2 quality dimensions were likely to be perceived as lower quality by the public. In comparison, the component scores for patient experience (*P*<.001), mortality (*P*<.001), and the new combined timeliness and effectiveness of care (*P*<.001) of the revised HCSR2021 are all positively associated with Google star ratings (as shown in Table 2, column 4). Moreover, the association with readmission, which was previously negative, has become statistically insignificant (*P*=.52). Variations in the HCSR component score on safety of care, either before or after the revisions, do not significantly affect Google star ratings (*P*=.49 and *P*=.30).

**Figure 2:**
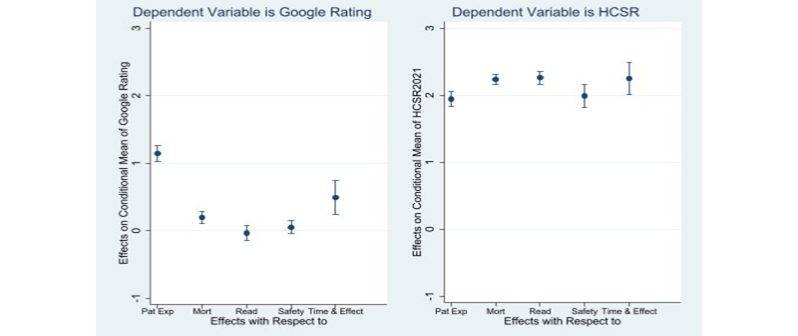
Average marginal effects of individual quality components of Hospital Compare Star Ratings (HCSR) issued by Centers for Medicare & Medicaid Services in April 2021 (HCSR2021) on Google star ratings and HCSR2021. Pat Exp: patient experience; Mort: mortality; Read: readmission; Safety: safety of care; Time & Effect: timeliness & effectiveness of care.

The coefficient estimates in Table 2 are difficult to interpret directly because the FRM estimation technique is nonlinear, the scaling of Google star ratings is between 0 and 1, and there are adjustments needed to account for different weightings of individual components in the final HCSR. Therefore, we calculated the average marginal effects of individual quality components and their 95% confidence intervals on Google star ratings and HCSR2021 (Figure 2; data source: Table 2, columns 3 and 4). Average marginal effect is the average of the marginal effects computed for every observation in the sample [22].

The average marginal effect of patient experience in the Google star ratings is 1.143 (*P*<.001), that is, 1 unit increase in patient experience scores of HCSR is expected to increase the Google star ratings by 1.143, on average. Similarly, the average marginal effect of mortality is 0.195 (*P*<.001), readmission is –0.035 (*P*=.52), safety of care is 0.051 (*P*=.30), and timeliness and effectiveness of care is 0.489 (*P*<.001). In comparison, the average marginal effects of all dimensions of quality on HCSR are statistically significant (*P*<.001). The average marginal effects of patient experience, mortality, readmission, safety of care, and timeliness and effectiveness of care are 1.942, 2.236, 2.260, 1.988, and 2.249, respectively.

**Table 1:** Fractional response probit regression of Google star ratings on HCSR^a^2020 and HCSR2021.

Variable	Google star rating^b^	*P* value	Google star rating^c^	*P* value
HCSR	0.076	<.001	0.070	<.001
Log beds	0.022	.005	0.009	.23
For-profit	0.165	<.001	0.165	<.001
Teaching	0.069	<.001	0.076	<.001
For-profit × teaching	0.007	.85	0.010	.79
Rural	–0.004	.81	0.009	.59
Rural × for-profit	–0.143	<.001	–0.155	<.001
Rural × teaching	–0.058	.04	–0.073	.009
Rural × for-profit × teaching	–0.037	.62	–0.045	.56
Constant	–0.015	.44	–0.005	.81
Observations	2619	N/A^d^	2619	N/A
Pseudo *R*-squared	0.005	N/A	0.004	N/A

^a^HCSR: Hospital Compare Star Ratings.

^b^This column reports estimation results using Google star ratings as a dependent variable with HCSR2020 and hospital controls.

^c^This column reports estimation results using Google star ratings as a dependent variable with HCSR2021 and hospital controls.

^d^N/A: not applicable.

**Table 2:** Fractional response probit regression of Google star ratings and HCSR^a^ on individual component scores before and after the change in methodology of computing HCSR.

Variables	(1) HCSR2020	*P* value	(2) Google star ratings	*P* value	(3) HCSR2021	*P* value	(4) Google star ratings	*P* value
Patient experience	1.376	<.001	0.640	<.001	1.249	<.001	0.600	<.001
Mortality	1.377	<.001	0.112	<.001	1.438	<.001	0.102	<.001
Readmission	1.378	<.001	–0.058	.01	1.454	<.001	–0.018	.52
Safety of care	1.272	<.001	0.016	.49	1.279	<.001	0.027	.30
Efficient use of medical imaging	1.370	<.001	–0.276	.06	—^b^	—	—	—
Timeliness of care	0.924	<.001	0.336	.07	—	—	—	—
Effectiveness of care	1.720	<.001	1.241	<.001	—	—	—	—
Timeliness and effectiveness of care	—	—	—	—	1.446	<.001	0.257	<.001
Log beds	0.005	.52	0.046	<.001	0.091	<.001	0.055	<.001
For-profit	–0.015	.39	0.201	<.001	–0.054	.007	0.199	<.001
Teaching	0.002	.90	0.067	<.001	–0.044	.005	0.060	<.001
For-profit × teaching	0.019	.49	–0.026	.45	0.009	.76	0.001	.98
Rural	–0.008	.55	–0.022	.14	–0.110	<.001	–0.029	.05
Rural × for-profit	–0.009	.74	–0.155	<.001	0.027	.47	–0.148	<.001
Rural × teaching	–0.001	.97	–0.056	.03	0.108	<.001	–0.042	.12
Rural × for-profit × teaching	0.063	.39	0.036	.60	0.036	.58	–0.005	.94
Constant	0.480	<.001	0.241	<.001	0.594	<.001	0.247	<.001
Observations	2619	—	2619	—	2619	—	2619	—
Pseudo *R*-squared	0.166	—	0.009	—	0.154	—	0.008	—

^a^HCSR: Hospital Compare Star Ratings.

^b^Not applicable.

## Discussion

### Principal Findings

Our analysis adds to the stream of prior studies analyzing the associations between social media ratings and HCSR or Hospital Consumer Assessment of Healthcare Providers and Systems (HCAHPS) ratings and other selected measures of clinical quality [3]. A recent systematic literature review identified 32 peer reviewed studies (25 using US data) published before 2020, which quantitatively examined relations between web-based ratings and health care outcomes [18]. These include studies by Bardach et al [14] and Ranard et al [15], which find a significant correlation between Yelp hospital ratings and HCAHPS survey scores of patient experience, and studies by Campbell and Li [16] and Huppetz and Otto [23], which find a positive association between Facebook star ratings and HCAHPS scores. Hawkins et al [24] find a positive association between the percentage of patients with top HCAHPS scores (>9) and the sentiment score derived from text analysis of Twitter posts. Subsequent studies also report similar findings about associations between web-based ratings (Yelp and Facebook) and HCSR and HCAHPS scores [17,19].

We refined those analyses by examining the associations between Google star ratings and HCSR and its individual quality components before and after the 2021 revision of HCSR methodology, and by using a more appropriate statistical estimation technique. We used Google star ratings because many studies report that patients find providers by first “googling” their symptom and then “googling” providers that care for conditions associated with their symptoms, suggesting that Google is increasingly used by consumers to search for and access care [7-9]. However, only one prior study has analyzed Google star ratings [19].

Our correlation analysis yields statistically significant positive correlations between crowdsourced Google star ratings and HCSR, confirming that Google star ratings provide directional information that is consistent with the expert HCSR. However, the narrower distribution of Google star ratings indicates that patients tend to avoid extreme ratings in web-based reviews, resulting in relatively lower Google star ratings for highest quality hospitals and vice versa. To the extent that web-based ratings influence hospital choices at the margin, the lower variance of Google star ratings may result in a relative underchoosing of highest-quality hospitals and overchoosing of lower-quality hospitals. FRM estimation with hospital type and size controls supports our conjecture that hospital-level characteristics such as size and type affect consumer perceptions of hospital quality and therefore web-based ratings. Estimation results in Table 1 (columns 1 and 2) show statistically significant positive coefficients on for-profit and teaching (*P*<.001), indicating that these hospitals receive higher Google star ratings compared to the baseline of urban, nonprofit, and nonteaching hospitals, even after controlling for the differences in hospital quality as measured by HCSR. Similarly, the negative coefficients on interaction terms rural × for-profit (*P*<.001) and rural × teaching (*P*=.04 and *P*=.009) indicate that these hospitals receive relatively lower ratings. The statistically significant positive coefficient on log beds (*P*=.005) in Table 1 (column 1) suggests that larger hospitals received higher Google star ratings even after controlling for the differences in hospital quality and type. However, with the revised HCSR, this bias in Google star ratings favoring large hospitals appears to have been attenuated (*P*=.23; Table 1, column 2).

Consistent with the findings in most prior studies that web-based ratings are associated with patient experience HCAHPS quality scores [14-16,23,24], our FRM estimates of the relationship between HCSR and its individual components in Table 2 show a positive association between Google star ratings and HCSR patient experience scores. Google star ratings also exhibit positive associations with component scores for mortality, timeliness of care, and effectiveness of care in HCSR2020 and the combined score for timeliness and effectiveness of care in HCSR2021, which suggests that patients are able to evaluate other quality dimensions at least partially. For example, the average time spent in the emergency room, an underlying measure of timeliness of care, and the percent of newborns whose deliveries were scheduled too early when medically not necessary—a measure used in assessing effectiveness of care, can be assessed by patients who usually do not possess medical expertise. The HCSR2020 quality scores for readmission and efficient use of medical imaging were negatively associated with Google star ratings, potentially leading to suboptimal choices by consumers of hospitals based on their perceptions regarding readmission and efficient use of medical imaging. The updated HCSR2021 does not provide a separate score for medical imaging, and the prior negative association between Google star ratings and readmission has become statistically insignificant, suggesting that the revised HCSR methods may partially ameliorate suboptimal hospital choices based on Google star ratings from previous rating methods. All together, these results suggest that crowdsourced Google star ratings have directional information value that is consistent with expert ratings on select dimensions of hospital quality.

A comparison of the marginal effects of individual components of quality on Google star ratings and HCSR (Figure 2) reveals a consistent pattern of underweighting of medical quality by patients who provide Google star ratings. As mentioned before, the overall variance of Google star ratings is less than the variance in HCSR because patients appear to avoid extreme ratings, which in turn explains the relatively lower sensitivity of Google star ratings to improvements in the component scores compared to HCSR. Moreover, because patients are not exposed to or are unable to assess the quality measures underlying readmission and safety of care, Google star ratings appear to be unaffected by the improvements in these quality components. In other words, hospitals need to make significant improvements in patient experience, mortality, as well as timeliness and effectiveness of care to improve their Google star ratings but should not expect improvements in safety of care and readmission to result in improved Google star ratings.

### Limitations

Our observations are subject to several caveats. Obviously, consumer choice of hospitals is a very complex decision guided not simply by summary ratings of hospital quality but by medical condition, financial situation, urgency, insurance status, network access, hospital location, and provider preferences. While our study does not analyze how web-based reviews affect actual consumer hospital choices, we draw on other studies [7,8,21], which show that hospital choices are influenced by web-based ratings and conjecture their marginal effects on hospital choices. Our analysis is based on a single source of web-based ratings and expert ratings in the United States and may not be generalizable across other countries and rating sources. We analyzed the associations between aggregate Google star ratings and HCSR and its component scores; however, we overlooked the potentially rich data contained in detailed web-based review comments. Additionally, Google star ratings are cumulative averages, while HCSR is based on the previous 3 years of data, which may result in mismatched data and lags in responsiveness. Greater transparency from Google about its algorithms would enable better comparability. Even the revised HCSR method has been criticized for the following reasons: using relative performance–based ratings instead of predefined standards of performance, lack of true peer group comparisons and risk adjustment, inadequate audit or verification of data, and dependence on advisory technical expert panels instead of rigorous peer review [13]. Future revisions to HCSR methods to address these issues will necessitate an updated analyses of associations of web-based ratings with HCSR.

### Implications for Research and Practice

Consistent with previous findings, our analysis shows that aggregate Google star ratings provide directional information consistent with the expert HCSR and are also associated with selected HCSR quality component scores related to patient experience and other components that patients can partially assess (eg, mortality as well as timeliness and effectiveness of care). While our results suggest even the aggregate web-based ratings are informative, future research employing natural language processing and sentiment analyses techniques on detailed web-based review comments and assessing their associations with quality measures in HCSR can generate more nuanced insights. Research is needed to analyze the causes of the observed divergence between web-based ratings and specific components of expert ratings. Hospital rating agencies such as CMS need to launch education efforts to address these knowledge gaps and to increase consumers’ use of expert ratings. Hospitals can benefit from using crowdsourced ratings as timely, accessible, and dynamic indicators of their quality performance, while keeping in mind their sensitivities and biases. Because of their universal and timely availability, crowdsourced data along with personal health applications, such as Google Health and Microsoft HealthVault, and electronic medical records will continue to evolve as important components of the larger “digital transformation” of health care [25]. Research to assess the interactive effects of these on decision-making by patients as well as health care providers is crucial.

## Abbreviations

CMS: Center for Medicare & Medicaid Services
FRM: Fractional Response Model
HCAHPS: Hospital Consumer Assessment of Healthcare Providers and Systems
HCSR: Hospital Compare Star Ratings
